# Detection of vector-borne pathogens in owned dogs with cranial cruciate ligament rupture living in the Mediterranean area

**DOI:** 10.1186/s13071-022-05205-x

**Published:** 2022-05-10

**Authors:** María-Dolores Tabar, Javier Tabar, Carolina Naranjo, Laura Altet, Xavier Roura

**Affiliations:** 1 Hospital Veterinario San Vicente Vetsum, Calle del Veterinario Manuel Isidro Rodríguez García Nº17, San Vicente del Raspeig, 03690 Alicante, Spain; 2 IDEXX Laboratories, Carrer del Plom 2, 08038 Barcelona, Spain; 3 Vetgenomics, Parc de Recerca Universitat Autònoma de Barcelona, Edifici Eureka, 08193 Bellaterra, Spain; 4 Hospital Clínic Veterinari, Universitat Autònoma de Barcelona, Carrer de l´Hospital s/n, 08193 Bellaterra, Spain

**Keywords:** *Leishmania*, *Ehrlichia*, *Anaplasma*, *Bartonella*, Piroplasms, Filariae, Osteoarthritis

## Abstract

**Background:**

Cranial cruciate ligament rupture (CCLR) results from a multifactorial degenerative process that leads to rupture of the ligament. Vector-borne pathogens (VBP) in dogs can induce joint disease but their role in CCLR has not been previously investigated. The aim of the present work is to evaluate the prevalence of VBP in dogs with CCLR.

**Methods:**

This was a prospective study that included 46 dogs presented for CCLR surgical treatment and 16 control dogs euthanized for diseases unrelated to the joints. Specimens collected included blood, synovial fluid, and synovial membrane biopsy. Pathogen testing consisted of serology for *Leishmania infantum* (quantitative ELISA), *Ehrlichia canis/ewingii*, *Borrelia burgdorferi*, *Anaplasma phagocytophilum*/*platys*, and *Dirofilaria immitis* (4DX IDEXX test), and PCR for *L. infantum*, *Ehrlichia*/*Anaplasma* spp., *Bartonella* spp., piroplasms (*Babesia* spp. and *Theileria* spp.), and filariae (*D. immitis*, *Dirofilaria repens*, *Acanthocheilonema dracunculoides*, *Acanthocheilonema reconditum,* and *Cercopithifilaria* spp.) on both EDTA-whole blood (EB) and synovial fluid (SF) samples. SF cytology and histopathological evaluation of synovial membrane were also performed.

**Results:**

The prevalence of VBP was 19.6% in the CCLR group and 18.8% in the control group, with no statistical difference among them. The presence of synovitis was not more frequent in CCLR dogs (45.6%) than in control dogs (43.7%). Lymphoplasmacytic infiltration was the most common inflammatory pattern detected in the joints of both groups of dogs.

**Conclusions:**

This study failed to demonstrate a role of canine VBP in CCLR or the presence or different pattern of joint inflammation in pathogen-positive dogs.

**Graphical Abstract:**

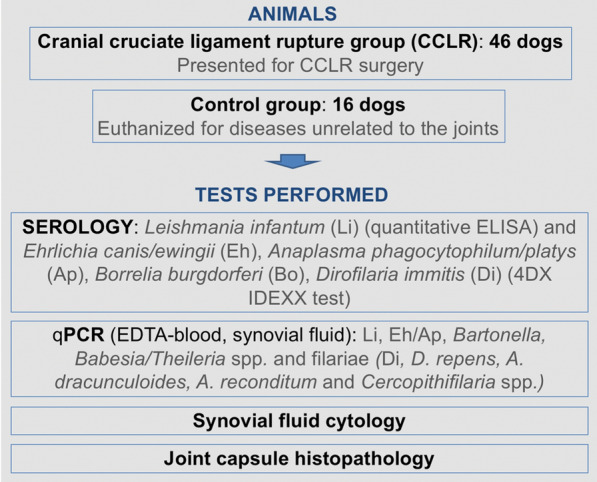

**Supplementary Information:**

The online version contains supplementary material available at 10.1186/s13071-022-05205-x.

## Background

Cranial cruciate ligament rupture (CCLR) is a common cause of pelvic limb lameness in dogs that results most often from a degenerative process that leads to rupture of the ligament. Several risk factors have been described, such as age, breed, sex, neutering status, and weight [1, 2]. However, CCLR is likely to have a multifactorial origin involving genetics, anatomic conformation, and chronic joint inflammation, eventually leading to rupture of the ligament and osteoarthritis [3, 4]. It has been suggested that immunopathological mechanisms may be involved in the development of degenerative CCL lesions [5] and, furthermore, lymphoplasmacytic synovitis is a common finding in dogs with CCLR [6]. Therefore, two hypotheses have been proposed regarding the role of chronic synovitis in the development of cruciate ligament fiber damage and, consequently, CCLR. The first is that synovitis is an early and primary event inducing progressive ligament fiber disruption [7]. The second is that several intrinsic factors may induce minor fiber ruptures with subsequent induction of chronic synovitis that itself contributes to the degenerative changes in the ligament, culminating in CCLR [7].

Previous publications reported an increased bacterial load in synovial membrane biopsies of inflamed stifles of dogs with CCLR in comparison with healthy stifles, suggesting that environmental bacteria can induce persistent chronic synovitis [8, 9]. Other kinds of pathogens such as vector-borne pathogens (VBP) can induce acute and chronic joint disease in dogs, but their role in CCLR has not been thoroughly investigated [10–14]. A recent study performed in Brazil reported that 91.3% of 46 dogs with leishmaniasis presented joint abnormalities observed on physical examination, radiography, and/or computed tomography, but CCLR was not included in the list of joint anomalies described [14]. Other VBP such as *Ehrlichia canis*, *Anaplasma phagocytophilum*, *Rickettsia rickettsii*, *Borrelia burgdorferi*, *Babesia canis*, *Bartonella vinsonii* subsp. *berkhoffii*, and filariae have been also linked to joint disease, such as polyarthritis, in dogs [4, 10, 15–24].

This study aims to detect several VBP in owned dogs with CCLR, to determine if there is an association between the presence of VBP and CCLR, and to identify the presence of a specific inflammatory pattern in the synovial membrane of dogs with CCLR and VBP.

## Methods

### Patient selection

This prospective cohort study was conducted from June 2016 until October 2019 in a referral veterinary hospital located in the Mediterranean basin. Forty-six dogs presented for CCLR surgical treatment and 16 control dogs that were humanely euthanized for suffering from different serious diseases that did not affect the joints, were prospectively included during that period.

Complete information obtained for the 46 dogs included age, sex, breed, body weight, general and orthopedic examination, location of CCLR (unilateral or bilateral), and pre-anesthetic urine and blood analysis. Furthermore, clinical reports for all dogs included in this study were reviewed to look for diagnosis of vector-borne disease, before surgery or euthanasia, or at any time after surgery until the end of the study (follow-up ranged from 1.5 to 4.5 years). Owner consent was received for all dogs prior to their enrolment in the study. This study was carried out in accordance with ethical guidelines of the International Council for Laboratory Animal Science.

### Sampling

Blood was collected by cephalic or jugular venipuncture from all dogs. Synovial fluid (SF) samples were collected immediately prior to the surgery or euthanasia, following anesthetic induction and aseptic preparation of the limb. Smears were made immediately after collection, obtained following cytocentrifugation, and 0.5 ml of synovial fluid was placed in a tube containing calcium ethylenediaminetetraacetic acid (EDTA).

Incisional synovial membrane biopsy specimens were obtained from the affected joint of dogs with CCLR or from a random stifle joint of control dogs following caudomedial parapatellar arthrotomy (1 cm sample from each dog). Synovial biopsy samples were fixed in neutral buffered 10% formalin and routinely processed for histologic examination.

Serum samples were archived and used for the detection and quantification of organism-specific serology. Both EDTA-blood and EDTA-SF samples from all dogs were stored frozen for posterior polymerase chain reaction (PCR) analysis. All serology and PCR analysis were performed at the same time at the end of the study.

### Serology

A quantitative enzyme-linked immunosorbent assay (LEISCAN^®^ enzyme-linked immunosorbent assay [ELISA]) was performed for *Leishmania infantum* antibody detection (IDEXX Barcelona, Spain), which has a cut-off of 0.55, with values > 0.55 considered positive. A commercial qualitative assay kit (SNAP 4DX Plus IDEXX, Hoofddorp, Netherlands) was employed for the detection of *E. canis*/*ewingii*, *B. burgdorferi*, and *A. phagocytophilum*/*platys* antibodies, and *D. immitis* antigen.

### DNA extraction and PCR amplification

DNA was extracted from 400 µl of both blood and SF samples as previously described [25]. Collected samples, previously defined as specific pathogen-free by PCR, were used as an extraction negative control in each extraction batch. Samples were tested in an operator blind manner, and piroplasms (*Babesia* spp. and *Theileria* spp.), *Bartonella* spp., *Ehrlichia*/*Anaplasma* spp., and filariae [*D. immitis*, *Dirofilaria repens*, *Acanthocheilonema dracunculoides*, *Acanthocheilonema reconditum*, and *Cercopithifilaria* spp.] were targeted using genus-specific PCR assays [26]. Briefly, real-time PCR was carried out in a total volume of 20 μl containing SYBR^®^ Select Master Mix (Thermo Fisher Scientific), specific primer, and 4 μl of 1/5 diluted DNA. The thermal cycling profile was 50 °C for 2 min and 95 °C for 15 s and 60 °C for 1 min, and a dissociation curve added at the end of the run. Water was used as a PCR negative control and commercial DNAs as positive PCR control. Quantitative *L. infantum* PCR was performed as described by Francino et al*.* [25]. *Leishmania infantum* DNA load was classified as very low, low, medium, high, or very high load as reported by Martínez et al. [27]. The eukaryotic 18S ribosomal ribonucleic acid (rRNA) (Thermo Fisher Scientific) was used as an endogenous control to ensure proper DNA extraction.

### Sequencing

In all cases in which SF or blood samples were PCR-positive, direct DNA sequencing was performed to characterize pathogens at the species level. Sequencing was carried out using the BigDye^®^ Terminator v3.1 Cycle Sequencing Kit (Thermo Fisher Scientific) following the manufacturer instructions and with the same primer used in the PCR and sequences compared with the GenBank database (https://blast.ncbi.nlm.nih.gov/Blast.cgi).

### Cytologic and histologic examination

Synovial fluid smears, obtained following cytocentrifugation, were stained with a rapid modified Romanowsky stain (QUICK PANOPTIC, Química Clínica Aplicada, Amposta, Spain), and light microscopy examination was performed only to detect the presence of pathogens.

Hematoxylin and eosin-stained biopsies of synovial membranes were evaluated by light microscopy. Samples were evaluated for the presence and pattern of inflammatory cells and characterized as lymphoplasmacytic, granulomatous, neutrophilic, or mixed. Dogs were considered to have synovitis if more than one inflammatory cell/high-power field (hpf) was identified.

### Statistical analysis

Statistical analysis was performed using commercially available statistical software (IBM SPSS Statistics v.19). Values for the prevalence of VBP (positivity to any of them) and for the presence of inflammation in joint capsule biopsy were established. Contingency table analysis was performed. Chi-square test and Fisher’s exact test were used to compare proportions of positivity, and statistical significance was set at *P*-value ≤ 0.05.

## Results

This study included 62 dogs, 46 with CCLR and 16 controls. Among CCLR dogs there were 25 females (17 intact and eight spayed) and 21 males (15 intact and six spayed), that ranged from 6 months to 11 years in age. There were 16 different breeds, with the most common being mongrel dogs (*n* = 18). In the control group there were eight females (four intact and four spayed) and eight males (six intact and two spayed) that ranged from 4 to 17 years in age, and 10 different breeds (Additional file 1: Table S1). Reasons for euthanasia included neoplasia (five), chronic renal failure (two), gastric dilatation-volvulus (one), pituitary hyperadrenocorticism (one), discal hernia (two), urethral obstruction (one), cardiac failure (two), refractory epilepsy (one), and acute liver failure (one). There was no previous clinical history, clinical signs, or clinicopathological abnormalities consistent with VBP infection in any of the dogs included in this study.

The prevalence of VBP in the CCLR group was 19.6% (9/46). *Leishmania infantum* was detected in six dogs, with three seropositive, one SF-PCR-positive, and two both seropositive and SF-PCR-positive. *Ehrlichia* spp. were found in three dogs, with two seropositive and one seropositive and blood-PCR-positive (*E. canis*). One of those dogs was co-infected with *L. infantum* (SF-PCR-positive) and *Ehrlichia* spp. (seropositive). Finally, *Theileria equi* DNA was detected in the SF sample of one dog (Additional file 2: Table S2).

Among the control group, the prevalence of canine VBP was 18.8% (3/16). All three dogs were positive for *L. infantum*, with one seropositive, one blood-PCR-positive, and one seropositive and both blood- and SF-PCR-positive (Additional file 2: Table S2).

No VBP were detected by light microscopy in any SF-smear of any dog included in this study, and the total prevalence of VBP was not statistically different between dogs with CCLR and control dogs [odds ratio (OR) = 0.949, 95% confidence interval (CI) 0.22–4.05, *P* = 0.629].

Unilateral CCLR was detected in 29 dogs (five of them with VBP), while CCLR was bilateral in 17 dogs (four of them with VBP), meaning that the presence of bilateral CCLR was not statistically more frequent among dogs with VBP (OR = 0.677, 95% CI 0.15–2.97, *P* = 0.439).

Histopathology of biopsies of synovial membranes yielded synovitis in 45.6% (21/46) of dogs with CCLR, with various inflammatory patterns including lymphoplasmacytic (17), neutrophilic (one), granulomatous (one), and mixed lymphoplasmacytic and granulomatous (two); and in 43.7% (7/16) of control dogs, all of them with lymphoplasmacytic infiltration. Presence of synovitis was not statistically more frequent in dogs with CCLR compared with control dogs (*χ*^2^ = 0.017, *df* = 1, *P* = 0.895) or in dogs with or without VBP (*χ*^2^ = 0.141, *df* = 1, *P* = 0.708). Furthermore, a different or specific inflammatory pattern was not detected among dogs with VBP, independently of whether they had CCLR or were control dogs (Additional file 2: Table S2).

When the outcome of VBP-positive dogs with CCLR was reviewed, two of three dogs with *L. infantum* SF-PCR-positive, but without previous clinical history or diagnosis of *Leishmania* infection, developed clinical signs consistent with patent leishmaniasis between 9 to 12 months after CCLR surgery (Additional file 2: Table S2).

## Discussion

This study failed to demonstrate a role of VBP in CCLR or the presence of a specific pattern of joint inflammation in VBP-positive dogs, even though several canine vector-borne diseases have been associated with joint damage [4, 10, 14, 16–22, 24], and some of them, especially leishmaniasis, are considered endemic in the area where the present study was performed [28]. Larger case–control studies would likely be needed to clarify the role of various vector-borne organisms as a cause or cofactor in the development of CCLR.

In canine leishmaniasis, the frequency of orthopedic problems had been reported to range from 44.8% to as high as 91.3%, when both orthopedic examination and imaging (radiology and/or computed tomography) were combined to look for joint abnormalities [14, 29]. Some anomalies found in the orthopedic examination include joint stiffness, lameness, soft tissue swelling, joint pain or crepitation, and functional disability. Notably, dogs with CCLR can have one or more of these described orthopedic abnormalities, but in previous studies there was no specific information about the prevalence of CCLR in dogs suffering from leishmaniasis. Theoretically, lameness in leishmaniasis could be produced by polyarthritis, with additional bone or muscle involvement, usually secondary to inflammation associated with the deposition of immune complexes within the joint because of a type III hypersensitivity reaction [4, 12, 30]. However, primary joint infection could also occur, and parasites have been identified within macrophages by cytologic evaluation of the synovial fluid and by histologic assessment of the synovial membranes [31, 32]. Thus, infected dogs could present with monoarthritis, oligoarthritis, or polyarthritis [18], and some reports indicate that stifle joint could be affected in close to 80% of cases [14]. In this study, *L. infantum* was the most frequent VBP detected in dogs with CCLR, although its prevalence was not significantly different from that in control dogs, suggesting no role of *Leishmania* infection in the pathogenesis of CCLR. A potential explanation for its detection in both groups of dogs could be the high prevalence of subclinical infection present in an endemic area of leishmaniasis [28, 33].

An association of polyarthritis with ehrlichiosis has been reported previously; however, there was no firm evidence to support it, and other possible co-infections were not ruled out, meaning that the relationship was controversial [10, 15–17]. In the present study, three dogs with CCLR were found to have *Ehrlichia* antibodies or DNA. However, in the two only seropositive dogs, infection could not be confirmed, and perhaps it could simply reflect exposure or past infection. The third dog, seropositive and *E. canis* PCR-positive, never developed any other clinical sign or laboratory abnormality consistent with patent or subclinical ehrlichiosis, either before or after CCLR surgery. This could imply that this dog perhaps was in an acute stage of the disease and could have recovered alone, or that it was in a subclinical stage. Either of these two scenarios probably rules out a relationship between CCLR and *Ehrlichia* infection.

*Theileria equi* is one of the equine piroplasms which is enzootic in Spain, with almost half of the horses having antibodies or circulating parasitemia [34]. This parasite has occasionally been detected in dogs, although its epidemiological and clinical significance remains unknown [35]. All the above, together with the fact that the dog in this study with *T. equi* in SF did not demonstrate any other clinicopathological abnormalities throughout the study period, could suggest that this pathogen was opportunistic without clinical significance on CCLR.

Although *Bartonella*, *A. phagocytophilum*, *B. burgdorferi*, filariae, or other piroplasms such as *Babesia* have been associated with acute or chronic canine polyarthritis [10, 13, 17, 19–22, 36], no dog in this study was positive for any of them. Those findings could be in concordance with the local geographic prevalence of those VBP reported in previous studies in the area evaluated in this study [16, 27, 34, 37]. However, it is worth noting that the limitations regarding the sensitivity of the techniques used and the limitations associated with the specimens collected for testing could also have contributed to failure to detect these organisms.

Lymphoplasmacytic arthritis was the most frequent histopathological finding in this study, in dogs both with and without CCLR. This agrees with previous publications where lymphoplasmacytic synovitis has been commonly described in dogs with CCLR [6], but it has also been detected in post-mortem samples from dogs without CCLR [7]. On the other hand, reactive immune-mediated arthritis due to deposition of developed immune complexes secondary to VBP infection is predominately neutrophilic [4, 11, 12, 18, 29]. This fact reinforces the idea that VBP did not play any role in the pathogenesis of CCLR, together with the fact that neither inflammatory pattern nor frequency of synovitis was statistically different between dogs with or without CCLR or between dogs with or without VBP in this study. However, although not statistically significant, three dogs with CCLR in this study showed granulomatous synovitis, a kind of inflammation also reported in several tissues in dogs with leishmaniasis [33, 38], but only one of them was positive for leishmaniasis. Although granulomatous inflammation is usually associated with the presence of *Leishmania* within the tissue [12, 13], amastigotes could not be detected in this seropositive and *Leishmania* PCR-positive dog with granulomatous synovitis. The cause of granulomatous inflammation in the other two dogs remains undetermined. Therefore, the current study could not definitely rule out a potential role of this VBP in the pathogenesis of synovitis and perhaps of CCLR in some of these dogs.

This study has some limitations. The low number of dogs included, due to the difficulty in recruiting cases due to the strict inclusion criteria and the fact that it was a prospective study with a control group, means that the statistical results must be taken with caution. Moreover, only dogs that received CCLR surgical treatment were included, excluding dogs initially diagnosed with VBP and CCLR that ultimately did not undergo surgery. An additional limitation was the wide variety of diseases that led to the euthanasia of control dogs and the fact that articular surfaces were not evaluated, especially knowing that the control population had a higher mean age. However, previous medical histories were extensively evaluated to exclude previous infections with VBP or diseases that could affect the joints. Furthermore, control dogs were also included during the same period and area as CCLR dogs, thus limiting the bias in the probabilities of VBP detection. Finally, the last limitation was the identification of the presence of VBP. In this study we used serology, microscopy in SF cytology, and synovial membrane biopsy, as well as PCR in blood and SF, seeking to maximize the chances of VBP detection. However, the positive serologies were not conclusive of causality of the abnormalities found in the joint. Moreover, it was not possible to perform serology for all the pathogens sought for, nor could immunohistochemistry and/or PCR on the biopsy be performed to increase the probability of detection of selected VBP.

## Conclusions

This study failed to demonstrate a role of several VBP in the pathogenesis of CCLR in dogs, or the presence or a different pattern of joint inflammation in pathogen-positive dogs. However, to overcome the limitations of this study, additional studies may be warranted to clarify the potential relationship between VBP and CCLR in dogs.

## Supplementary Information

**Additional file 1: Table S1.** Distribution of breeds among the study groups.

**Additional file 2: Table S2. **Dogs with vector-borne pathogens detected (*NA* no abnormalities; *LP *lymphoplasmacytic; *GR* granulomatous; *NIAD* no infectious agent detected).

## Data Availability

The datasets used and/or analyzed during the current study are available from the corresponding author on reasonable request.

## Abbreviations

CCLR: Cranial cruciate ligament rupture
VBP: Vector-borne pathogens
*D*: *Dirofilaria*
*A*: *Acanthocheilonema*
EB: EDTA-blood
SF: Synovial fluid
*E*: *Ehrlichia*
*L*: *Leishmania*
