# Supplementary material for: Detection of vector-borne pathogens in owned dogs with cranial cruciate ligament rupture living in the Mediterranean area
Source: Parasit Vectors. 2022 May 10;15:105. doi: 10.1186/s13071-022-05205-x (PMC9088045; doi:10.1186/s13071-022-05205-x)
Supplement: Supplementary file 1 — Additional file 1: Table S1. Distribution of breeds among the study groups. [file 13071_2022_5205_MOESM1_ESM.docx]

Table S1. Number of dogs and breed distribution among the study groups

| **Group** | **Breed** |
| --- | --- |
| Control  (16) | Mongrel (4), Labrador Retriever (3), Yorkshire Terrier (2), Beagle (1), Bodeguero (1), Poodle (1), Chihuahua (1), Dalmatian (1), Shar-pei (1), West Highland White Terrier (1) |
| CCLR  (46) | Mongrel (18), American Staffordshire (4), Labrador Retriever (4), Pit Bull (4), Boxer (3), Mastiff (2), Yorkshire Terrier (2), Spaniel Breton (1), Great Dane (1), Bull Mastiff (1), Golden Retriever (1), Italian Mastiff (1), German Shepherd (1), Rottweiler (1), Hound (1), Shar-pei (1) |
