# Supplementary material for: Detection of vector-borne pathogens in owned dogs with cranial cruciate ligament rupture living in the Mediterranean area
Source: Parasit Vectors. 2022 May 10;15:105. doi: 10.1186/s13071-022-05205-x (PMC9088045; doi:10.1186/s13071-022-05205-x)
Supplement: Supplementary file 2 — Additional file 2: Table S2. Dogs with vector-borne pathogens detected (NA no abnormalities; LP lymphoplasmacytic; GR granulomatous; NIAD no infectious agent detected). [file 13071_2022_5205_MOESM2_ESM.docx]

Table S2. Dogs with vector-borne pathogens detected

| **Case** | **Group** | ***Leishmania* serology (Positive ≥ 0.55)** | ***Leishmania* blood-PCR** | ***Leishmania* SF-PCR** | ***Ehrlichia***  **serology** | ***Ehrlichia***  **blood-PCR** | ***Theileria* SF-PCR** | **Synovial biopsy** | **Previous diagnosis of leishmaniosis** | **Patent leishmaniosis developed during follow-up** |
| --- | --- | --- | --- | --- | --- | --- | --- | --- | --- | --- |
| 3 | CCLR | **0.67** | - | - | - | - | - | LP synovitis | No | No |
| 4 | CCLR | **1.42** | - | **Medium load** | - | - | - | LP + GR synovitis | No | **Yes** |
| 12 | CCLR | - | - | **Medium load** | **+** | - | - | LP synovitis | No | No |
| 22 | CCLR | - | - | - | **+** | ***E. canis*** | - | LP synovitis | No | No |
| 32 | CCLR | - | - | - | **+** | *-* | - | LP synovitis | No | No |
| 35 | CCLR | **1.02** | - | **Low load** | - | *-* | - | NA | No | **Yes** |
| 43 | CCLR | **1.63** | - | - | - | *-* | - | NA | Yes | Yes (relapse 1 year after treatment was discontinued) |
| 50 | CCLR | - | - | - | - | *-* | ***T. equi*** | LP synovitis | No | No |
| 64 | CCLR | **1.18** | - | - | - | *-* | *-* | NA | Yes | No |
| 48 | Control | **1.02** | - | - | - | *-* | *-* | NA | No | No |
| 55 | Control | - | **Medium load** | - | - | *-* | *-* | NA | No | No |
| 57 | Control | **1.14** | **Very high load** | **Medium load** | - | *-* | *-* | NA | No | No |

(NA= no abnormalities; LP= lymphoplasmacytic; GR= granulomatous; SF=synovial fluid)
